# Digital transformation: A meta-review and guidelines for future research

**DOI:** 10.1016/j.heliyon.2023.e12834

**Published:** 2023-01-07

**Authors:** João Reis, Nuno Melão

**Affiliations:** a Industrial Engineering and Management, Faculty of Engineering, Lusofona University and EIGeS, Campo Grande, 1749-024, Lisbon, Portugal; b CISeD–Research Center in Digital Services, Polytechnic Institute of Viseu, Campus Politécnico, 3504-510, Viseu, Portugal

**Keywords:** Digital transformation, Meta-review, PRISMA, Organizations, Social, Technologies, Sustainability, Smart cities

## Abstract

The emergence of digital transformation has changed the business landscape for the foreseeable future. As scholars advance their understanding and digital transformation begins to gain maturity, it becomes necessary to develop a synthesis to create solid foundations. To do so, significant steps need to be taken to critically, rigorously, and transparently examine the existing literature. Therefore, this article uses a meta-review with the support of the Preferred Reporting Items for Systematic Reviews and Meta-Analysis (PRISMA) Protocol. As a result, we identified six dimensions and seventeen categories related to digital transformation. The organizational, technological, and social dimensions are still pivotal in digital transformation, while two new dimensions (sustainability and smart cities) still need to be explored in the existing literature. The need to deepen knowledge in digital transformation and refine the dimensions found is of paramount importance, as it involves some complexity due to organizational dynamics and the development of new technologies. It was also possible to identify opportunities, challenges, and future directions.

## 1 Introduction

In recent years, academics have provided in-depth knowledge regarding Digital Transformation (DT). These contributions were carried out in the production industry [1], service industry [2], healthcare [3], and education [4], just to name a few areas. However, these studies are dispersed across several academic fields. As the academic community realized this limitation, researchers became interested in gaining a broader view of DT through systematic literature reviews (SLR) within each field [5–7] and some of them about the DT phenomenon itself [8]. Although the aforementioned works have contributed to significant advances in knowledge, there are no records of articles providing a detailed holistic view of DT. To fill this gap in the literature, we followed the suggestions of notable scholars [9,10] and set out to undertake a meta-review. Along with this, we also identified reports of other phenomena about DT, such as the paradox of digital technologies [11]. If, on the one hand, there is a belief in the benefits of adopting DT, on the other hand, there has been some frustration with DT and its impacts on organizations. Conceptually, DT benefits organizations with better operational efficiency [6,12], greater innovation [13], and cost reduction [14] in the medium-long term. However, the implementation of DT is complex as it entails initial costs, requires changes, and creates resistance from workers [15]. Therefore, DT adoption may be risky without models and tools that assist its implementation across organizations. Viewed in isolation, this meta-review may be considered ambitious; however, it can become a relevant work if viewed from a holistic perspective, along with other systematic reviews. We opted for a meta-review because it can ensure reproducibility and transparency of the entire review process. To this end, we explained the methodological process in detail and included the content analysis process (see AppendixA) to make the entire process visible to readers. With DT changing rapidly, the need to identify opportunities, challenges, and future directions is critical. In this regard, we developed the following research question: What are the drivers of DT promoting scientific growth? The answer to the previous question can be achieved by addressing the following objectives: (1) identifying the most relevant thematic areas; (2) categorize the literature on DT; and (3) propose future research based on recent studies. We consider this study original and innovative because it fills an important gap in the literature. In November 22nd, 2022, after performing a search on Elsevier Scopus with the search terms “digital transforming” and “meta-review” in the title of the document, no result was found; in title-abstract-keyword only four documents were found, but they were not directly related to the theme. These results obtained in one of the most important international databases are surprising, especially considering the exponential growth of research on DT in recent years.

The next section provides a conceptualization of DT and associated terms. We then explain the PRISMA process and how the data was collected and analyzed. The results section presents a holistic theoretical-conceptual model of DT and a research agenda. Finally, the conclusions section focuses on managerial, theoretical, and original contributions.

## 2 Conceptual overview

In the existing literature, concepts referring to DT are still inconsistent or treated simplistically [16,17]. Although there is still some difficulty in accepting a consensual definition of DT, this section describes the relationship between digitation, digitalization, and DT. If it was common to find conceptual miscellanea in the past between digitization, digitalization, and DT, this issue now seems to be overcome. In that regard, Kohli & Johnson [18] stress that digitization is commonly associated with transforming traditional processes into digital ones. Loske & Klumpp [19] also consider that digitization is a “process of converting analog data into digital data sets.” Furthermore, recent research argues that digitization encodes or shifts analog tasks and information into a digital format so that computers can store, process, or transmit information without altering value-creating activities [20]. An excellent example of digitization is e-books or downloadable music, i.e., converting tangible products into products delivered digitally [18].

Digitalization, in turn, is described as digital technologies that can be used to alter existing business processes. In that regard, companies are investing in products and process innovation through new digital solutions, allowing them to deal with more data and information [21]. One example is the creation of online or mobile communication channels allowing customers to connect with companies more conveniently than through traditional interactions [22]. Thus, within the scope of digitalization, companies must apply digital technologies that allow the optimization of existing business processes, i.e., better coordination between processes and creating value for the customer. In short, the difference between digitation and digitalization lies in creating value and improving the customer experience.

Although the concept of DT has gained significant notoriety only recently, it dates back to the 90's [23]. DT goes beyond digitalization as it involves changing organizational processes and tasks, which typically lead to developing new business models [17]. Thus, DT consists of integrating information technologies in companies' operations, whether internal or external [24]. It can also be considered as a change that occurs with the implementation of technologies in a system within a company [19]. This transformation is supported by the adoption of new technologies from which new performance, new processes, and new business models emerge [25,26]. In addition, DT is not only linked to technology, but also to an improvement in the business model, collaboration, and culture [27]. This transformation arises with the use of digital tools in the daily activities and processes of the company, being subsequently achieved through its promotion inside and outside it [28]. For instance, DT can be employed in several domains, such as the healthcare sector; in this regard, the wide and deep use of information technologies changes how health services are delivered and processed [29]. A company that opts for DT seeks to offer a product and/or service through new digital formats, thus achieving a link between physical processes and virtual processes [23]. Some authors identify several possible contributions of DT in a company, such as: (1) optimization of physical and digital resources; (2) obtaining greater competitive advantage; (3) greater creation of value for the customer; and (4) cost reduction [30,31].

However, not all industries have been able to keep up with this technological pace and adopt digital technologies, either due to investment difficulties or lack of adaptation of their business model [32, p. 141]. In a digital company, success involves accepting market uncertainty and volatility, identifying opportunities and having the ambition to realize them, as well as making quick decisions taking into account innovation, customers and competitors [33]. DT has played a disruptive role in various sectors of activity. However, the retail sector was considered one of the sectors most prone to DT [30,32]. This is due to the emergence of new consumers called “digital natives”, who have driven the use of digital platforms and, consequently, the need for innovation in current business models [7]. The next section discusses the data collection process, the content analysis, and the research limitations.

## 3 Materials and methods

**Fig. 1:**
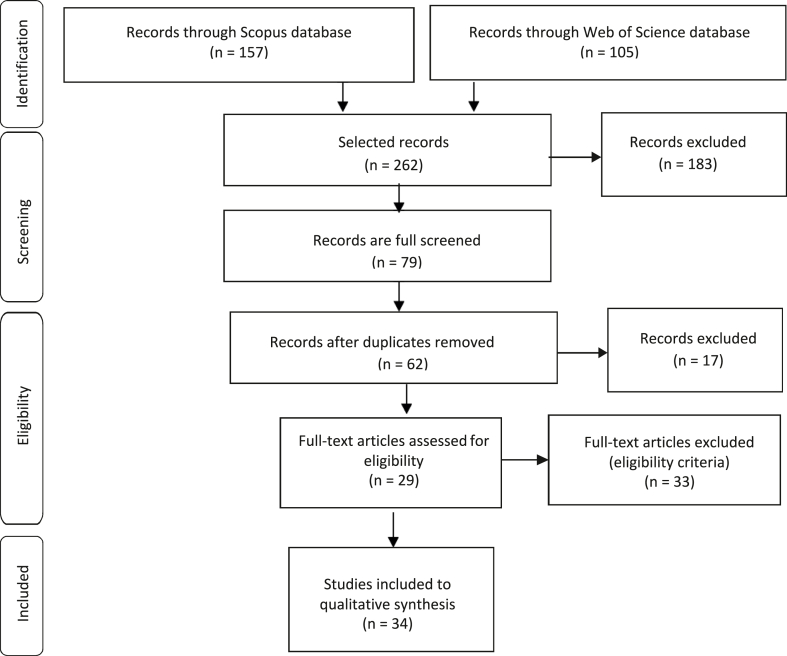
PRISMA flowchart.

This article uses a meta-review, as it aims to synthesize the existing body of completed and recorded work produced by researchers [34]. Meta-reviews are methods known to be able to gather the literature and which can have a significant influence on research, practice, and policy [35]. A Preferred Reporting Items for Systematic Reviews and Meta-analysis (PRISMA) also supports the meta-review to discover new ideas, concepts, and debates in a critical, rigorous, and transparent way. PRISMA included a checklist of 27 items and four-phase flowchart (Fig. 1), enabling data extraction from two of the largest abstract and citation databases of peer-reviewed literature.

The search was conducted in Elsevier's Scopus and Web of Science Core Collection (WoS) on December 8, 2021 (Fig. 1). This search combined the terms “digital transformation” and “systematic literature review” in the Title-Abstract-Keywords (TITLE-ABS-KEY) to identify the manuscripts within the area of research (identification phase). Then, we applied pre-selected filters (i.e., language, source, and document type) to identify the most relevant manuscripts (screening phase). The next phase included accessibility criteria (eligibility phase), which encompassed removing duplicated articles and those that were not strictly related to the topic. Finally, articles not identified in the Scopus and WoS databases were included (inclusion phase). Incorporating additional articles allowed to justify and/or reinforce the arguments used in the results section. That is, highly cited conference papers in DT [17] can also be relevant and should not be left out. We were careful with the issue of transparency, and, for that reason, we included the flowchart (Fig. 1) and their respective explanation. As mentioned earlier, data collection in the Scopus and WoS databases was carried out until the end of 2021. Both databases were selected because they are considered the largest international and multidisciplinary research databases of peer-reviewed manuscripts. This argument is also used by researchers who have published articles on DT in top-tier Journals, such as Benavides et al. [5] and Lombardi and Secundo [36], or in some cases, just one of the selected databases, such as the WoS, by Zhu [8], and Scopus by de Bem Machado et al. [37]. A more objective argument that justifies using Scopus and WoS is related to the coverage of journals in the area of Natural Sciences and Engineering [38], areas typically associated with DT. Moreover, we could have much broader data coverage [39] and free access if we selected Google Scholar. However, despite being a powerful search engine, it does not guarantee that the documents included have been peer-reviewed.

After performing the search using the terms “digital transformation” and “systematic literature review” in the TITLE-ABS-KEY, we identified 262 manuscripts. Following this, we applied the filter by full-text journal articles to obtain high-quality research articles. For readability and interpretation reasons, we selected only articles in English; otherwise, difficulties in interpretation could lead to biased results. This phase resulted in the selection of 79 scientific journal articles. The eligibility phase allowed the elimination of 17 duplicate articles and 33 articles that did not correspond to the research objectives, resulting in 29 articles. The last phase included 5 more articles, so in the end, we were left with 34 articles to analyze. The PRISMA protocol we followed uses the same process of identification, screening, eligibility, and inclusion of other relevant scientific articles published in Q1 journals, whose databases were Scopus and WoS [40].

Data were encoded twice. First, the articles were manually encoded. That is, the articles were read in full, and repeated words and text excerpts were identified (AppendixA). Data analysis was performed using low-tech material (e.g., Excel). However, as a significant number of articles were being examined, text analysis using a computer-assisted data analysis package is recommended. Therefore, the second step included using NVivo12 [41], a qualitative data analysis software for researchers. Data were analyzed using the content analysis technique [42]. This technique allowed coding the most important phrases and words [43], making it possible to identify patterns in emerging codes and ideas. Specifically, the process was carried out in four stages: first, we read the entire texts to identify the most relevant phrases and ideas, followed by a coding process; second, we associated excerpts/codes from the selected articles with the categories and added new ones as necessary; third, we identified emerging patterns and ideas (dimensions); lastly, we revised the previous categories, making adjustments, until redundancies and contradictions were clarified and the results were easily interpreted. In short, this technique enabled to code and analyze a large volume of data. After the content analysis, we also followed a verification process: first, we compared the two analyzes, the aforementioned manual cross-analysis with NVivo12; secondly, a verification that included the analysis of the articles’ keywords. The latter step included cross-checking the categories and sub-categories (i.e., our manual categorization) with the 34 articles’ keyword statistics (i.e., authors' choice) and which can be retrieved directly from Scopus. This process allowed to identify discrepancies in the data analysis. As we found similarities, we consolidated the coding process.

Despite the advantages of meta-review, this methodology also has limitations. Applying filters may have excluded relevant documents from other databases (PubMed, etc.), search engines (e.g., Google scholar), or other forms of publication (e.g., books, chapters). However, the PRISMA technique has an advantage over traditional systematic reviews because, unlike the latter, PRISMA (last phase) allows the inclusion of relevant articles overcoming the aforementioned limitation. Lastly, this article presents a “snapshot” of the reality, as both databases are permanently being updated.

## 4 Results and discussion

### 4.1 Digital transformation overview – influential topics and subject areas

**Fig. 2:**
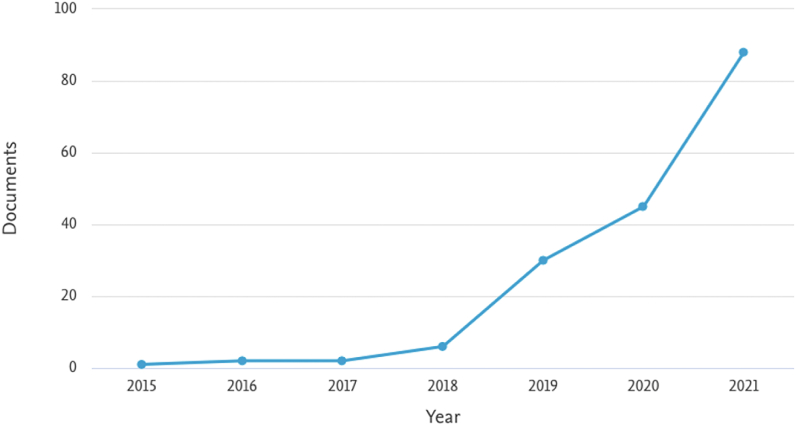
Documents by year (retrieved from Elsevier Scopus).

**Fig. 3:**
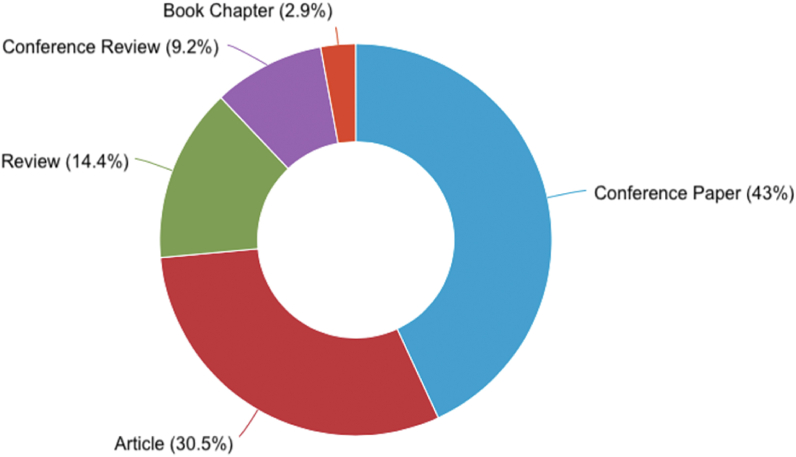
Documents by type (retrieved from Elsevier Scopus).

This section aims to respond to the first research objective. To transparently identify the most relevant thematic areas, we use the graphs provided directly by the Scopus database, which is the leading database for this article (similarly used by Lombardi and Secundo [36]). Compared to WoS, Scopus was selected for covering a wider range of journals, both in keyword search and citation analysis [16]. Additionally, most papers indexed in WoS are included in Scopus [44]. Indeed, when we exclude repeated articles (i.e., screening phase, Fig. 1), most of the selected articles come from Scopus. Therefore, for this section, the first initial terms “digital transformation” and “systematic literature review” were used in the Scopus TITLE-ABS-KEY (resulting in 157 articles), which allowed us to identify the most relevant thematic areas. This graphical analysis aims to provide the most holistic view possible in order to provide readers with an overview of the results. For example, from this analysis, the reader can easily infer that the topic is growing exponentially (Fig. 2) and that only 30% of Scopus documents have been analyzed (Fig. 3). For quality reasons, the content analysis had to focus only on journal articles, being therefore more restricted.

Fig. 2 shows the upward scientific interest in DT, especially from 2018 onwards. This phenomenon is probably explained by the maturity of the topic, making it possible to analyze the existing literature with some relevance. In particular, we can see that published studies have mainly focused on business model strategies [45–49], digital business [48,50,51], the use of disruptive technologies [47,[52], [53], [54]], sustainability [55,56], human resources [57–59], and smart cities [45]. In turn, Fig. 3 shows the types of documents focused on DT. The publication in conference proceedings is an indicator that DT is arousing the interest of researchers in the scope of the discussion of ideas and the search for solid knowledge on the subject. In terms of article publishing, we have seen that the appetite of top-tier indexed Journals is high, as 45% of the articles are from Q1 Journals and 31% from Q2 Journals.

**Fig. 4:**
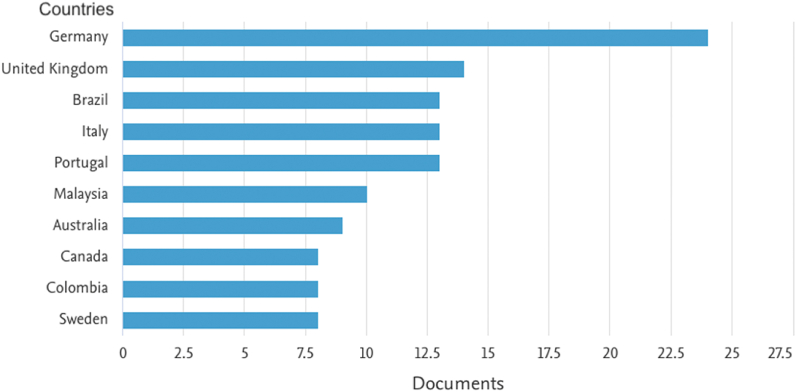
Documents by country or territory (retrieved from Elsevier Scopus).

Regarding the distribution of papers by country, we can see that Germany, the United Kingdom, and Brazil are the ones that stood out the most (Fig. 4). Germany stands out from the other economies, as German industry is one of the main drivers of Industry 4.0 (I4.0). To do so, Germany has made a significant investment in research, which is essential for initiatives aimed at digitizing the manufacturing industry [56]. For instance, Siemens has formed a research alliance in industrial automation and digitization with the state-funded Technical University of Munich, the Ludwig-Maximilians University, the German Research Center for Artificial Intelligence, and the Fraunhofer Institute for Applied and Integrated Security Applications [60].

**Fig. 5:**
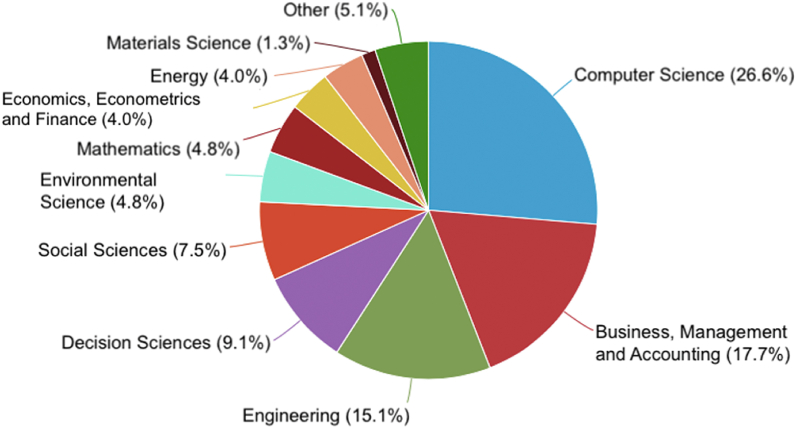
Documents by subject area (retrieved from Elsevier Scopus).

Considering that one of the drivers of the German economy has been I4.0, it is not surprising that the areas with the greatest scientific research are computer science (26.6%) and engineering (15.1%) in the context of the development of cyber-physical systems, cybersecurity, cloud computing, advanced robotics, just to name a few. Fig. 5, with no surprise, also includes the subject area of business, management, and accounting (17.7%), given the impact of its coverage in different countries, industries, companies, and people. In that regard, Kraus et al. [61] argue that DT has led to considerable changes in many organizations, no longer seen as just a technological opportunity but as a way to introduce new processes that can improve the main structures of how companies do business.

### 4.2 Digital transformation overview – dimensions and categories

This section presents a general view of the existing literature regarding DT, thus responding to the second research objective. We focused exclusively on the analysis of the 34 articles that were selected from Scopus and WoS (Fig. 1). Table 1 shows the dimensions and categories identified during data analysis. AppendixA presents a series of tables with more detailed information (including codes/phrases). Although it is not common to see tables with the complete content analysis available in scientific articles, we decided to make all the information available to the reader for transparency and reproducibility reasons.

**Table 1:**
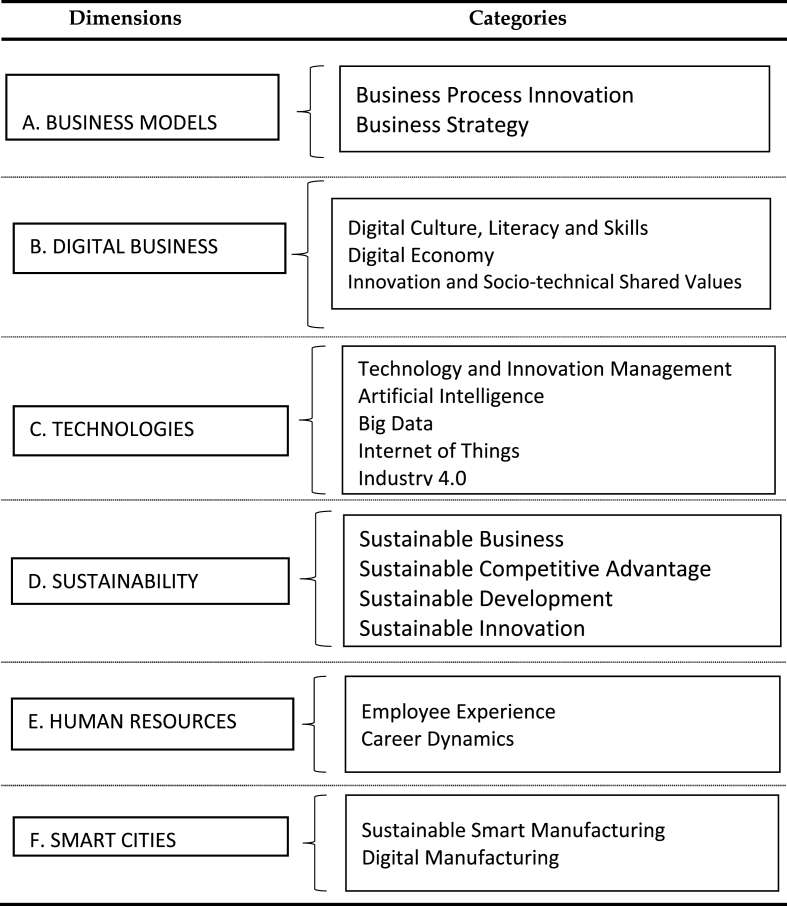
Dimensions and categories.

#### 4.2.1 Business models

The first dimension addresses (but not limited to) topics, such as (Table A1): (1) business process innovation, which is improving the competitive position of organizations [45,54] and bringing disruptive DT to the global industrial value chain [53,60,62]; (2) digital business strategy that enhances productivity [46,63,64] and creates new value for customers [65].

With regard to innovation, the trend is for organizations in DT environments to implement value-added innovation by integrating social and economic dimensions from different types of innovation, such as product-service and process innovation, as well as innovation in business and organizational models [54,60]. Developing a digital business strategy is critical for organizations as DT involves business and technology issues, transcending organizational boundaries [46]. Furthermore, selecting technologies (i.e., tech-oriented) is vital to the business strategy and can significantly add value to the business [63]. Initially, Information Technology (IT) strategy was seen as a functional- and secondary-level strategy component; however, nowadays, DT is the central pillar of the strategy, driving the emergence of the “digital strategy” concept [48]. Thus, in the context of the digital age, the organizational environment is also more volatile, uncertain, complex, and ambiguous (VUCA), so the rapid changes in competition, demand, technology, and regulations are more challenging than ever. In that regard, the pressure on companies to align their business strategy with the changing technological environment has increased significantly with the emergence and growing importance of new disruptive digital technologies [60,64]. Therefore, a digital business strategy demands strong leadership, an agile and scalable core, and a clear focus on customer engagement or a digitized solutions strategy [65]. The “tech-oriented” view fails to capture the more fundamentally important role of the “procedural” character of DT, demanding a deeper and more complete “transformational” effort on vision, strategy, culture, human skills, resources and infrastructures, business model, and company's competitiveness [48,61].

In short, with regard to business models, we found that process innovation is changing the business landscape, increasing competitiveness through the development of new digital services and products. In that regard, the business strategy focuses on disruptive technologies. The VUCA environment pushes for a more comprehensive and transformational strategy where people and resources adapt to organizational needs.

#### 4.2.2 Digital business

The second dimension addresses (but not limited to) topics, such as (Table A2): (1) digital culture, literacy, and digital skills that are enhancing DT efforts [52,58,64]; (2) digital economy and the challenge of measuring the potential generated by digital technologies [65,66]; (3) innovation and socio-technological shared values, being seen as an opportunity to balance the responsibilities assigned to humans and machines [54,65].

When it comes to digital business, organizations wanting to benefit from their technology investments need to strengthen the digital skills of their workforce [58]. Therefore, the workforce is one of the key actors in transforming the organization, as digitally capable human resources will be managing and using technology [48,66]. Furthermore, employees working in digitally mature organizations describe their culture as more collaborative and innovative than traditional ones [64].

The success of the digital economy is expected to be ensured by strengthening the position of companies through the quality of corporate governance and financial structure, aligned with the latest technologies. The digital economy is seen as an economy that accelerates the DT of existing economic sectors, promotes new ecosystems enabled by digital technologies, and develops a digital industry [66]. Thus, the digital economy includes a combination of digital infrastructure, socio-technical processes, and information and communication technologies [56]. The risk of the digital economy is associated with the large-scale acceleration of the development of new technologies, which seems almost unstoppable due to the intensive innovation trend. Moreover, recent studies have also stressed that the greatest challenge many organizations face when investing in DT is finding a way for equating, reimagining and redefining the employees experience and bringing their digital literacy up to date. At this level, artificial intelligence (AI) is demanding greater skill in terms of problem solving, as it begins to outperform human performance in executing analytically complex cognitive tasks. Thus, the challenges appear to be twofold, both from the point of view of technological acceleration and the digital literacy of the workforce.

#### 4.2.3 Technologies

The third dimension addresses (but not limited to) topics, such as (Table A3): (1) technology and innovation management, which has been one of the main drivers of DT [48,52,61,64,65,67]; (2) AI and big data, which have been propelling significant developments in carrying out analytical-cognitive activities both in organizations and in the industry [55,56,58,62,64,68]; and the (3) Internet of Things (IoT) and I4.0, which involves the interconnection of computing power and intelligent data flow, enabling process control in the service and production industry [48,62].

Technology is one of the main drivers of DT, giving a significant boost to organizations that integrate this key factor into their strategy [62]. As mentioned earlier, technology is an enabler of DT that is causing a change in value creation, as it supports the development of new business models and a focus on acquiring new skills and competencies [67]. One of the largest consultancies, McKinsey & Company, proposed a model based on six building blocks that allows implementing a successful end-to-end transformation for industrial companies. These six blocks naturally go beyond the simple technology upgrade and are: (1) Create a business-led technology roadmap; (2) Talent development and qualification; (3) Adopt an agile delivery methodology; (4) Moving to a modern technology environment; (5) Focus on enriching data management; (6) Conduct the adaptation and scaling of digital initiatives [52]. With regard to technology, DT has aroused interest in specific digital technologies, such as AI and big data [65]. Due to VUCA pressure, companies are aligning their business strategy with digital technological change (e.g., AI, Big Data) [64]. In that regard, AI is defined as the transformation of service-product processes into automated processes, dependent on intelligent computer systems or robots that do not require human intervention to perform tasks associated with intelligence [6,47]. Despite the well-known advantages of AI and robotics, current discussion often covers the risks of automation. Debates have focused more on the adaptability of jobs in DT than on replacing human labor [69]. Most studies suggest that complex socioemotional tasks continue to be performed by human beings, while cognitive-analytic tasks will be increasingly migrated to machines [70]. DT has therefore led to the formation of the digital organization, whose most volatile asset is AI and computational capital, evidenced in the continuous growth of automated information and the creation of digital products [56]. Digital technologies such as AI, big data analytics, and social platforms generate positive improvements for society (smart cities) and industry (I4.0) [55]. Thus, DT has been described as the change in an organization's structure, processes, functions and business models due to the adoption of digital technologies such as IoT, AI, machine learning, augmented reality, just to mention a few [17,58]. Therefore, DT does not focus only on organizations, but on almost all domains of knowledge, as it radically changes the concepts traditionally defined in organizational and management science [68].

#### 4.2.4 Sustainability

The fourth dimension addresses (but not limited to) topics, such as (Table A4): (1) sustainable businesses that focus on the integration of new and disruptive technologies [53,55,56]; (2) sustainable competitive advantage by integrating these technologies into the companies’ business processes [47]; (3) sustainable development with an emphasis on the United Nations Sustainable Development Goals (SDGs) [56]; and (4) sustainable innovation with an emphasis on open innovation theory [53].

Transformation to I4.0 has involved occupational adaptations to ensure quality and sustainable business models [56], leading to carbon emissions reductions [55] and an augmented degree of social responsibility [53]. Within the scope of DT, industry-specific IT resources are valued because they reduce costs, supporting sustainable competitive advantages as a result [62]. Therefore, the objective of companies is to establish sustainable performance and competitive advantage by integrating technology in the decision-making process with corporate strategy [47]. Additionally, the open innovation paradigm suggests that a holistic and cognitive approach to corporate governance, based on a regime of cooperation between internal and external resources for value creation, opens the possibility of redefining business models in which knowledge develops horizontally. This is achieved by involving all actors in the corporate ecosystem to gain a long-term sustainable competitive advantage [53]. The interest is in understanding and presenting the impact of digitization initiatives on economic growth and the achievement of the United Nations SDG [56].

#### 4.2.5 Human resources (HR)

The fifth dimension addresses (but not limited to) topics, such as (Table A5) employee experience, career dynamics, and type of human-machine relationships [57,58].

Within DT, HR concerns have been about the ability of employees to establish Human-Robot Interaction and Collaboration (HRI-C) relationships. At this level, the discussion is broad and involves a change in culture, mindset, and skills required from employees [58]. However, dealing with DT and the establishment of HRI-C dynamics can be challenging, particularly if employees are not ready for them. Therefore, the pressure to create HRI-Cs can create information overload and employee anxiety [58]. On top of that, while the benefits of a diverse workforce are well known, the career dynamics of individuals with technical differences over the rest are not well understood [57]. These different levels of expertise conflict with the balance between the professional and personal lives of the workforce. Therefore, companies must find strategies to balance professional and personal life for individuals who move to more specialized fields.

Furthermore, the literature also highlights that “a change management strategy to gradually change the mindset of the workforce and senior management, and instill the idea that there is no end to change” [52, p. 15]. It is recommended that organizations should develop change management models in DT environments, similar to traditional models (e.g., Lewin's or Kotter's change management models). In that regard, Attaran and Attaran [63] go further, stating that organizations fail to change because leaders do not pay enough attention to change management, which negatively affects the companies’ HR, making the next change more challenging to implement.

#### 4.2.6 Smart cities

The sixth dimension addresses (but not limited to) (Table A6) smart manufacturing [45,55,60], in particular the use of disruptive technologies to produce high-value products and services. Smart cities are not exactly smart manufacturing; however, smart manufacturing contributes to a larger scenario, acting as an enabler of smart cities. This aspect emerges from our analysis and is in line with the arguments of Suvarna et al. [71]. According to these authors, smart manufacturing contributes to smart cities not only from a technological point of view but also because it satisfies sustainability issues, which are important indices that make up a smart city. Other authors, such as Lom et al. [72], followed the same argument when they stated that process-based I4.0 with smart city transportation systems could create very effective, demand-driven, and highly productive manufacturing companies, while contributing to the sustainable development of society.

DT has attracted increasing interest from academics and practitioners regarding sustainability and intelligence/automation, such as smart cities, smart homes, smart governments, and smart production [45]. In particular, the alliance between sustainability and intelligence is at the center of academic discussion, highlighting themes such as sustainable smart manufacturing being enabled by digital technologies, such as IoT, cloud computing, big data, cyber-physical systems, AI, etc. [55]. These disruptive technologies have been offering unprecedented opportunities to create and develop value-added products and services [73]. In that regard, we identified that smart cities work as an extensive smart ecosystem, including different value activities and specific business functions and technologies [60]. To stimulate research on smart cities, there have been numerous special issues published by top-tier journals [73,74]. Thus, according to our analysis, smart cities are in increasing development, being a promising research area.

### 4.3 Proposed research agenda

The meta-review sets the stage for a research agenda. This review documents what is already known and, using critical knowledge gap analysis, helps to refine research questions, concepts, and theories to point the way for future research [75]. The articulation between the research question and the DT dimensions allowed the definition of the research agenda. Thus, the proposed research agenda defines the research areas and priorities that guide scholars.

Early in this article, we presented four figures that allowed us to identify the publication of documents by year (Fig. 2), type (Fig. 3), country (Fig. 4), and subject area (Fig. 5). The areas of research identified with the most remarkable growth are open innovation (Table A4. Sustainability) and I4.0 (Table A1. Business Model and Table A3. Technologies), within the scope of (1) Computer Science; (2) Business, Management, and Accounting; (3) Engineering (*vide* Fig. 5). An example that illustrates the scientific development of the areas above (i.e., open innovation and I4.0); is given by Savastano et al. [60], referring to the case of the alliance between Siemens with the state-funded Technical University of Munich, the German Research Center for Artificial Intelligence, and the Fraunhofer Institute for Applied and Integrated Security Applications.

Some topics described above were also identified in the content analysis stage (i.e., six dimensions and respective categories), allowing us to pinpoint the research priorities for DT. Below, the reader can find the main contributions of the article that frame the research agenda:

• According to the literature, VUCA environments are pushing for comprehensive and transformational digital strategies, changing the business landscape by increasing competitiveness in developing new services and products. To streamline research on the development of smart services and products, several special issues have been published by leading journals [76]. Therefore, disruptive technologies (AI, Big data, etc.) and innovation have been one of the main drivers of DT in building new digital services and products, and this trend is likely to continue.
• Compared with an early DT literature review, published in 2018 by Reis et al., new dimensions have been highlighted in this article. The three dimensions identified by Reis et al. [17] are still widely explored, namely organizational (Table A1 and A2), technological (Table A3), and social (Table A5). However, the new dimensions, namely sustainability (Table A4) and smart cities (Table A6) are still underdeveloped. What is new in this article is that while sustainability and smart cities are widely explored in other research domains (e.g., social sciences, engineering, etc.), within the scope of DT (i.e., business and management), it still falls far short of expectations. This argument may be also supported by a quick search in Elsevier Scopus (dated May 15th, 2022) with the keyword “sustainability” in TITLE-ABS-KEY, which indicates that the top 3 subject areas are Environmental Sciences (18.2%), Social Sciences (15.2%), and Engineering (11.3%); Business, Management, and Accounting represents only 7.5% of worldwide research. With regard to “smart cities”, a similar search shows that the top 3 subject areas are Computer Sciences (31.7%), Engineering (19.6%), and Social Sciences (11.2%); Business, Management, and Accounting represents only 2.6% of the worldwide research. This is a significant gap, considering that, in the scope of DT, the subject area Business, Management, and Accounting is in the top two with 17.7% (Fig. 5).
• From our analysis, future research may focus on the latter two dimensions (i.e., sustainability and smart cities). In that regard, researchers point out that empirical studies linking DT and sustainability are still scarce [77]. At the same time, recent growth in digital technologies is enabling cities to streamline smart services and offering new products [78]. This argument is also pointed out by some recent studies that have investigated the literature on DT in the context of meta-reviews Reis et al. [73] or meta-synthesis [79] in smart cities. Therefore, we argue that additional efforts are needed to reduce the knowledge gap between these two concepts (sustainability and smart cities) and DT.
• During data analysis, we tried to use the MECE rule (mutually exclusive and collectively exhaustive). MECE is a framework that allows solving complex problems by dividing them into sub-problems that are mutually exclusive (they do not overlap) and comprehensively exhaustive (cover all possibilities). The application of MECE rule was impossible in this context because of the difficulty of developing mutually exclusive sub-dimensions; nevertheless, the attempt presented interesting results. We delved deeper into this issue and realized that MECE is particularly important for creating taxonomies, as vague definitions cause overlaps between dimension characteristics [80]. An example is represented by the difficulty in the past in distinguishing between digitization, digitization, and DT. Since then, DT has been extensively investigated, with a clear conceptual distinction. But DT is so comprehensive that the concept crosses several research domains and dimensions (such as those identified in this article). For instance, the HR dimension is transversal to all other dimensions, such as technology (i.e., redefinition of HR skills) or digital business (sociotechnical values). In real terms, the dimensions identified are closely related to each other, covering all possibilities (i.e., comprehensively exhaustive). The MECE rule may still be used in the future, for mixed studies that incorporate literature review and empirical research for each of the dimensions identified in this article.
• Lastly, the research agenda includes the suggestion to analyze the impact of incorporating various technologies and how they can influence companies at different levels – individual, departmental, and organizational. In this regard, Kozanoglu and Abedin [58] argue that future studies could investigate one or several technologies to determine how their number and/or qualities can influence employees at an individual and company level. More specifically, they give the example of the article by Du et al. [81] that analyzes the use of blockchain in the business processes of a financial company.

In short, when answering the research question, we found six dimensions of DT, along with seventeen categories and sixty-six codes. Four dimensions, out of six, have already been explored in early reviews of DT literature [17]. Thus, this article is original insofar as we evidenced that “sustainability” dimension has been driven by open innovation in the context of improving new business models; and the “smart city” dimension has been driven by disruptive technologies in the context of the development of smart systems.

## 5 Conclusion

### 5.1 Theoretical contributions

To the best of our knowledge, this is the first time a meta-review on DT has been carried out. For that reason alone, this article is already original, bringing a timely contribution. From what we could extract from the analysis, there was a significant growth in literature reviews on the subject. Therefore, the academic interest in meta-reviews *per se* justifies publication. The article contributes to the theory as it provides clear guidance on research paths. The main contribution is, therefore, the definition of a research agenda focused on six dimensions, namely: 1) business models; 2) digital business; 3) technologies; 4) sustainability; 5) human resources; 6) smart cities. In that regard, we also provided the categories that emerged from the analysis, giving a clearer perspective of each dimension.

In general terms, it was possible to identify two new dimensions compared to previous studies – sustainability and smart cities. The existing literature points out that empirical studies link DT and sustainable business. While the most skeptical readers of this article might claim that sustainability is a widely explored dimension, it seems to fall short of expectations in the context of DT. In this context, sustainability has been driven by open innovation in terms of improving new business models. With regard to smart cities, the development of disruptive technologies has been the key driver of progress. It seems pertinent, thus, to reduce the knowledge gap on sustainability and smart cities in the context of DT.

### 5.2 Managerial contributions

With regard to managerial contributions, the results of this article are somewhat limited. First, because this article follows a literature review strategy; second, because the article's objective was to define a scientific agenda. Nevertheless, we were able to identify some contributions. In particular, it was possible to verify that due to the link between DT and technology, the significant areas of development are connected to computer sciences and engineering. Thus, for companies that intend to invest in DT, from the point of view of recruiting and training of HR, it may be helpful to consider investments in the areas of industrial engineering, computer engineering, and management. At the organizational level and in the context of the digital age, managers who intend to pursue a DT strategy should pay special attention to the open innovation ecosystem (e.g., n-Helix), rather than investing in company-centric innovation. From a business point of view, there are opportunities within the scope of smart cities that should be explored, namely in developing new technologies and sustainable development.

### 5.3 Original contributions

According to the results of the meta-review, we found that the most relevant concern is the need to reduce the gap regarding sustainability and smart cities in the context of DT. Crossing that gap in the literature and what is new and original in this article, we would like to highlight some frustration with the DT implementation, specifically with sustainable HR, a neglected dimension both empirically and theoretically. In that regard, the literature stresses that a change management strategy is essential to develop sustainable HR by instilling the idea that there is no end to change. Thus, organizations must develop management models for change in DT environments, similar to those traditional models that already exist, such as the ADKAR model or Kotter's change management model. The suggestion of developing new DT HR models is particularly relevant in digital business. Technological acceleration is forcing organizations to strengthen the digital skills of their workforce. The debates around adapting the workforce to DT contexts are not new. However, we advocate the development of HR sustainability models to adapt the workforce to Digital VUCA environments, where technological acceleration persists. Moreover, the existing literature refers the need to develop comprehensive transformational organizational efforts, particularly from a socio-technical perspective [48]. From our analysis, the smart cities dimension is very focused on smart production/manufacturing. Thus, in our view, the socio-technical approach is underdeveloped in this context. The same is not valid regarding the business model and digital model dimensions. We may have found our mutually exclusive sub-dimension in the sociotechnical issue. In other words, the socio-technical issue is a subset that still is not transversal to the different DT dimensions. However, as far as we know, there are already several articles outside the context of this research that analyze the socio-technical issue in smart cities [82,83] (although not focused on DT), which leads us to believe that a greater degree of scientific deepening is needed.

## Figures and Tables

**Table A1:** Business Models dimension

Dimension	Categories	Codes	Phrases/Excerpts
Business Models	Business Process Innovation	Multiple business Models	“Digital technologies have facilitated pervasive changes in business models, and some significant trends have emerged. However, the reconfigured business models are often not ‘new’ in the unprecedented sense. Business model innovations are primarily reflected in using digital technologies to enable the deployment of a wider range of business models than previously available to a firm. A significant emerging trend is the increasing adoption of multiple business models as a portfolio within one firm. This is happening in firms of all sizes, when one firm uses multiple business models to serve different markets segments, sell different products, or engage with multi-sided markets, or to use different business models over time.” [49]
		Knowledge Management	“Companies are able to direct their behavior towards innovative and sustainable business models, increasing the degree of social responsibility and obtaining a reputational advantage with the interested parties. Therefore, considering that knowledge is a critical resource for the company, it becomes interesting to understand how KMs, pushed by digital innovation, can accelerate the process of creating value in the long term, guiding the corporate strategy towards new, innovative business models.” [53]
		IT-Driven Changes	“Companies unable to rapidly develop and implement DT strategies and new digital business models are unlikely to keep pace and compete with the new digital reality. IT-driven changes enable business network-based value creation to become a feasible and valuable business model.” [62]
		IT-Enable Change	“The consequences of DT – such as the emergence of new digital business models even in non-digital industries – seem to extend beyond those of previous phases of IT-enabled change, which were usually related to the practice level and rather incremental change within firms. In summary and as indicated by recent works, it seems that the phenomenon of DT differs from past IT-related organizational change and cannot be explained entirely using established theoretical models. Instead, DT seems to have a more intricate and encompassing connection to the topic of organizational change, requiring a broader view of and comparison with the literature on organizational change and innovation.” [46]
		New Business Models	“Digital transformation has stimulated new business models and has caused disruptions in the global markets and industry. The shock waves of digital transformation have crashed the traditional businesses, resulting from the entry of digitally savvy firms.” [55]
		Transition Towards I4.0	“Organizations willing to seize the opportunities of I4.0 must thus innovate their processes and business models. The challenges that companies must face for the transition towards I4.0 paradigm are not trivial. Several digital transformation models and roadmaps have been lately proposed in the literature to support companies in such a transition. The literature on change management stresses that about 70% of change initiatives—independently of the aim—fail to achieve their goals due to the implementation of transformation programs that are affected by well-known mistakes or neglect some relevant aspects, such as lack of management support, lack of clearly defined and achievable objectives and poor communication.” [52]
		Transition Towards Market Needs	“To transform an industry, any technology needs to link the industry to an emerging market need through a business model. For example, when transitioning to a cloud-based business model, a software vendor found a new manner of consuming computer resources that provide advantages, such as low installation costs, no need for in-house servers, pay-as-you-go, great flexibility, and scalability.” [65]
		Disruptive Technologies	“Disruptive technologies are the bearer of radical changes in business models and ecosystems. Digital technologies, in particular, have led to major shifts in the industries that have adopted them.” [60]
		DT Conceptualization	“The concept of DT incorporates digital trends at different levels, including technology, processes, organizational aspects, especially business model disruption and society” [6]
		DT Conceptualization	“DT in a production setting covers the utilization of DT technologies described above in the manufacturing ecosystem. It targets the implementation of interconnecting, smart, and self-controlled structures of processes and systems, which will have implications for value creation, business models, downstream services and work organization, flexibility, optimized decision-making, resource productivity, and efficiency.” [45]
	Business Strategy	IT Strategy	“Instead of being viewed as a functional level and, in several situations, guided by a corporate strategy, the IT strategy should be incorporated into the organizational strategy in a systematic way named digital business strategy (or digital strategy), which comprises a corporate strategy developed and applied to leverage digital assets to achieve differential value. The usage of the next generation of AI technology in accordance with a well-defined digital business strategy, taking into consideration the company requirements, rules and automation, will create a competitive advantage for the business. Thus, it is also required to learn how executives can formulate competitive and cognitive strategies in order to innovate by leveraging the ability of the new age of AI. It is therefore necessary to examine human feelings, attitudes and requirements that fuel the motivations to interact with products and services centered on cognitive technologies.” [47]
		Digital Business Strategy	“DT requires an alignment of a company's multiple strategies to a digital business strategy combining both business strategy and IT. Other authors advocate that an independent DT strategy is essential.” [62]
		VUCA (Volatile, Uncertain, Complex and Ambiguous) Environment	“In the digital age, the environment of organizations is changing faster and has become more volatile, uncertain and complex than in the past. Rapid changes in competition, demand, technology and regulations make it more important than ever for organizations to be able to respond and adapt to their environment. In this context, the pressure on firms for aligning their business strategy with the technological changes in the environment has significantly increased with the emergence and growing importance of new digital technologies, such as Social Media, Cloud Computing, Big Data and Analytics, Embedded Devices, 3D-Printing, the Internet of Things, and Artificial Intelligence.” [64]
		Innovation Mechanisms	“For innovation mechanisms, we found that DT involves novelty in both strategic and operational regards. For instance, developing a digital business strategy is a key activity linked to strategy. It includes both business and technological aspects transcends organizational boundaries, and is based on data insights.” [46]
		IT Strategy	“On a firm level, IT Strategy was traditionally subordinated to the firms' primary business strategy, being merely an “embedded” (and secondary) functional-level strategy component, working as an “enabler” of the organizational capabilities. In contrast, today's DT relevancy and impact rest as the strategy's central pillar, driving the emergence of the “digital strategy” concept.” [48]
		Digital Workplace	“A digital workplace is the foundation for a successful business strategy – it enhances collaboration and leads to increased productivity.” [63]
		Digital Business Strategy	“A digital business strategy demands strong leadership, an agile and scalable core, and a clear focus on either a customer engagement or a digitized solutions strategy.” [65]

**Table A2:** Digital Business Ecosystems dimension

Dimension	Categories	Codes	Phrases/Excerpts
Digital Business	Digital Culture, Literacy and Sills	Digital Culture	“Attributes enhancing digital transformation efforts: risk-taking, test & learn, no-blame culture, customer centric, open to change, agile, autonomy of employees, …” [64]
		Digital Literacy	“Skills, knowledge and abilities of a person or social group used while interacting with digital technologies is described as employees' digital literacy, which is beyond traditional literacy perception limited purely to the ability to read, write and use printed texts in various contexts. Research previously has considered digital literacy of employees as a critical dynamic capability of organizations during their digital transformations. Recent studies have stressed that the greatest challenge in many organizations in digital transformation and innovation is finding a way for re-imagining the employees' experience and bringing their digital literacy up to date.” [58]
		Digital Skills	“The success of I4.0 initiatives depends on digital skills and knowledge that the company is able to recruit or train. So, the company should introduce new figures, like data scientists, user-interface designers or digital innovation managers, by recruiting new employees or training existing ones to put digitization into place. A specific step to analyze and define digital skills and capabilities is included in many consulting firms' DTMs, namely the PwC, Deloitte and McKinsey DTMs: these models highlight the essential role of digital skills in a successful digital transformation. They recommend that companies assess and map the digital capabilities needed to enable digital transformation and also suggest methods to create and acquire those capabilities that are not internally available; to this end, PwC's and McKinsey's DTMs suggest employee upskilling through dedicated digital training programs, as well as external recruitment to hire others' capabilities; Deloitte, beside the methods suggested by McKinsey and PwC, suggests the creation of partnership and recruitment as-a-service to access the needed capabilities.” [52]
	Digital Economy	Digital Technologies	“Digital technologies function as an enabler for more global, collaborative, and open activities. While expectations concerning the overall potential of digital technologies are high, measuring the digital economy's size and impact is challenging. In the digital world, firms face an environment in which constant connectivity allows for and demands more interactions and involvement of customers and collaborators and in which access to resources has often replaced their ownership.” [65]
		Digital Economy Definition	“A digital economy is an economy that accelerates the digital transformation of existing economic sectors, fosters new ecosystems enabled by digital technologies, and develops a next-generation digital industry in sectors with cybersecurity as an engine of growth.” [66]
	Innovation and Sociotechnical Shared Values	Knowledge	“Knowledge is the focus of the PSD framework, as it uses Innovation and Sociotechnical shared values to support Digital Transformation (DT) throughout New Product Development (NPD) projects. The sociotechnical approach regards DT environments as worldwide sociotechnical ecosystems, where systems are connected in networks, algorithms, people and industrial organizations.” [54]
		Sociotechnical Processes	“Digitalization refers to sociotechnical processes of digitization application at social and institutional levels. However, this digitalization concept differs from digitization in that the last refers to converting analogue sources into digital ones at narrow levels.” [56]

**Table A3:** Technological dimension

Dimension	Categories	Codes	Phrases/Excerpts
Technologies	Technology and Innovation Management	Technology	“It considers technology as a key driver of DT, and the different advantages that organizations and companies can obtain by introducing these techniques into their strategies and ways they operate. Considering how and when to apply them is and will remain a key factor.” [62]
		Digital Transformation Technologies	“The term digital transformation is a subject that is widely discussed among practitioners, but also paths its way as a scientific discipline. It affects industries, people and organizations. Technology is seen as a major driver and enabler of digital transformation. Those digital transformation technologies (DTT) cause changes in value creation. Companies adapt their strategies, explore new business models, and focus on acquiring new skills and competences. The major goals of digital transformation are increased flexibility, more customer-centric processes, and cutting costs.” [67]
		Building-Blocks	“Recently, McKinsey proposed a six-building-block model to help industrial companies implement a successful end-to-end transformation that goes far beyond simple technology upgrades. The six building blocks of the digital transformation are: (1) Creating a business-led technology road map; (2) Developing and up-skilling talent; (3) Adopting an agile delivery methodology; (4) Shifting to a modern technology environment; (5) Focus on data-management enrichment; (6) Driving the adaptation and scaling of digital initiatives.” [52]
		Innovation Management	“For innovation management, the ubiquity of data—if managed effectively—offers a valuable resource.” [65]
	Artificial Intelligence	Business Strategy	“In the digital age, the environment of organizations is changing faster and has become more volatile, uncertain and complex than in the past. Rapid changes in competition, demand, technology and regulations make it more important than ever for organizations to be able to respond and adapt to their environment. In this context, the pressure on firms for aligning their business strategy with the technological changes in the environment has significantly increased with the emergence and growing importance of new digital technologies, such as Social Media, Cloud Computing, Big Data and Analytics, Embedded Devices, 3D-Printing, the Internet of Things, and Artificial Intelligence. They are profoundly transforming the strategic context of organizations: changing the structure of competition, the behavior and expectations of customers, the way business is conducted, the way products are manufactured and services are delivered, the way of working and, ultimately, the nature of entire industries.” [64]
		Digital Technologies	“We show how the interest in particular digital technologies, such as artificial intelligence (AI) and blockchain, has drastically increased since 2017 and how the interest in all thematic fields has grown over time.” [65]
		Redefinition of Human-Resources Skills	Also, on the human “side” of firms, DT is playing a massive role in equating, rethinking, and redefining human-resources skills and capabilities, where new technologies like artificial intelligence (AI) are starting to surpass human performance, posing a social challenge on adapting talent to the new digital context, that demands higher levels of complexity, abstraction, and problem-solving skills [48].
		Transformation of Service Processes	“Artificial Intelligence (AI), understood as the transformation of service processes into automated processes that rely on intelligent computer systems or computer-controlled robots that do not require human intervention to execute tasks associated with intelligence.” [6]
		Jobs and Human Labor	“Some authors discuss the risk of automation among the advances in the field of artificial intelligence (AI) and robotics. According to them, economic and public debates on new technologies substituting for human labor are overestimated; they emphasize the adaptability of jobs in DT instead. In their study, they claim that the majority of jobs involve non-automatable tasks, and workers in highly exposed occupations (e.g., bookkeeping and accounting) perform tasks such as problem-solving or influencing that machines other- wise struggle with. Nevertheless, although the exposure of automation should be measured at the level of jobs rather than occupation, one in ten jobs are in fact susceptible to exposure.” [6]
		High Performance and Competitive Advantages	“In the digital age, businesses need reduced waiting periods and thus more awareness in the market environment that could alter faster than previous decades. Through this view, several organizations have been adopting emerging technologies designed to obtain high performance and a competitive advantage. Amongst these advancements, Artificial Intelligence (AI) has held a pivotal position and has drawn the attention of both researchers and the industrial sector. AI is referred to as the ability of a machine to learn from experience, adjust to new inputs and implement human-like tasks. AI could now be the innovation entity with the most significant potential for disruption. Likewise, AI is the fundamental multi-purpose technology in the domain, especially in relation to machine learning tools.” [47]
		Digital Organization	“In this sense, digital transformation leads to the formation of the digital organization, whose most volatile asset is the asset of artificial intelligence and computer capital, evidenced in the continuous growth of automated information and the creation of digital products.” [56]
		DT Conceptualization	“Digital transformation refers to the unprecedented disruptions in society, industry, and organizations stimulated by advances in digital technologies such as: artificial intelligence, big data analytics, cloud computing, and the Internet of Things (IoT).” [55]
		Positive Improvements	“Digital technologies such as artificial intelligence (AI), big data analytics, mobile technologies, IoT, and social platforms generate positive improvements for society and industry.” [55]
		Change in Organizations	“Digital transformation has been described as the change in an organization's structure, processes, functions and business models due to the adoption of digital technologies (such as Internet of things, artificial intelligence, machine learning, augmented reality, in-memory computing).” [58]
		Disruptions caused by COVID-19	“Digital technologies such as artificial intelligence and big data analytics will play a big role in dealing with disruptions caused by the pandemic, which requires uplifting employees' skills and literacy.” [58]
		Disruptive Technologies/Born Digital	“If the past few years are any indication; the next decade is likely to be more disruptive than the last. This is most evident in areas experiencing rapid technological change, especially artificial intelligence, cloud component engineering and born-digital service innovation such as disruptive FinTech.” [57]
		I4.0	“The digital transformation of not only organizations but of almost all areas of our lives radically changes the classically defined concepts in organizational and management science. It introduces completely new terms describing previously unknown phenomena (e.g. Cyber-Physical System, Industry 4.0, artificial intelligence, deep learning, big data, blockchain, e-commerce), and also expands the semantic fields of some terms that in “analog times” were used by organizational science.” [68]
	Big Data	Tech-Driven	“A good part of practitioners, namely firms' man- agers, consider that DT is mostly “tech-driven,” placing an excessive level of concern on new technological concepts as big data (BD), AI, internet of things (IoT), cloud computing (CC), social networks (SNs), blockchain, and others. This “tech-oriented” view fails to grasp the more fundamentally important role of DT's “processual” character, demanding a more profound and thorough “transformational” effort on firms' vision, strategy, structure, culture, human talent, resources and capabilities, business model, and competitiveness. Hence, still, many firms strive to react to DT's challenges adequately and end up following a “me-too” strategy model that adopts new technology concepts as a trend and not as a real business imperative, poorly allocating internal resources, and capabilities around technological “hypes and hopes,” while expecting good results.” [48]
		Big Data Conceptualization	“The concept of Big Data was used to describe the “volume, velocity and variety of data” that becomes increasingly difficult to analyze through conventional data processing tools. Currently, digital technologies enable homogenization and storing of significant amounts of data using big data analytics, or “advanced tools and techniques to store, process, and analyze the large volume of data.” [6]
		Interplay	“The increasing importance of the interplay between big data and DT affects business structures.” [62]
		General Purpose Technologies	“The major technological areas which enable DT are very diverse and traditionally called “general purpose technologies” These include, for example, cyber-physical systems (CPS), (industrial) internet of things (I/IoT), cloud computing (CC), big data (BD), artificial intelligence but also augmented and virtual reality.” [50]
		Smart Cities	“Many cities are increasingly adopting specialized digital technologies such as big data and IoT to address issues related to the environment and society. Digital technologies such as IoT infrastructure, cloud computing, big data, mobile Internet, and artificial intelligence are at the core of smart cities to enhance the environment, resources, and connectivity. These technologies provide unprecedented opportunities to combine sustainability principles in the context of smart cities and orchestrate strategies for fostering sustainable cities that aim to provide citizens with better services while reducing the footprint on the environment.” [55]
	Internet of Things (IoT)	DT Conceptualization	“Digital transformation has been described as the change in an organization's structure, processes, functions and business models due to the adoption of digital technologies (such as Internet of things, artificial intelligence, machine learning, augmented reality, in-memory computing).” [58]
		IoT Conceptualization	“IoT is explained as the network that connects through the internet and stipulates processes through information exchange and communications. IoT has become more relevant with the radical changes in the data and communication industry. Hence, mobile devices and technologies such as data management and cloud systems are significant components in IoT. The concept of IoT is composed of a mix of hardware and software technologies that connect individuals or groups. The term “things” in the concept of “internet of things” denotes either real or virtual actors or components in the network infrastructure. The connection is made via several modes such as 2G/3G/4G, global system for mobile communications (GSM), general packet radio service (GPRS), radio frequency identification (RFID), wireless fidelity (WIFI), global positioning system (GPS) and wireless sensor networks. The ultimate function of the IoT is to enable real-time information sharing with any of the autonomous network actors in the network. Patel and Patel classifies IoT into three categories, which are: (a) people to people, (b) people to machine/things and (c) things/machine to things/machine, linking through the internet.” [84]
	Industry 4.0	Industry 4.0 Concept	“Industry 4.0 concept evolves, new concepts arise, like “digital value chains, developing rich digital ecosystems gathering suppliers, manufacturers, and customers, changing both intra- and inter-firm logistics that facilitate the creation and emergence of virtual horizontally integrated value networks.” [48]
		Disruptive Digital-Enabler Concepts	Four disruptive digital-enabled concepts currently are supporting the DT of organizations: 1) The fourth industrial revolution, or Industry 4.0 (I4.0), which relates to “the systematic connection of technical compo- nents and processes [ …], supply and […] business relationships including all logistical elements”. It is based on the concept of I4.0, the Internet of Things (IoT), which describes the interconnection of computing power and data flows of smart objects that enable the autonomous control of daily life processes; 2) Artificial Intelligence (AI), understood as the transformation of service processes into automated processes that rely on intelligent computer systems or computer-controlled robots that do not require human intervention to execute tasks associated with intelligence. The concept of Big Data was used to describe the “volume, velocity and variety of data” that becomes increasingly difficult to analyze through conventional data processing tools. Currently, digital technologies enable homogenization and storing of significant amounts of data using big data analytics, or advanced tools and techniques to store, process, and analyze the large volume of data.” [6]

**Table A4:** Sustainability dimension

Dimension	Categories	Codes	Phrases/Excerpts
Sustainability	Sustainable Business	Sustainable Business Models	“Changes caused by the Fourth Industrial Revolution involve occupational adaptations in order to ensure quality and sustainable business models.” [56]
		Sustainable Business Practices	“Companies are now relying on AI, IoT, and big data analytics for carrying out sustainable business practices that involve reduced carbon emission and minimizing other waste to the environment.” [55]
		Innovative and Sustainable Business Models	“Companies are able to direct their behavior towards innovative and sustainable business models, increasing the degree of social responsibility and obtaining a reputational advantage with the interested parties.” [53]
	Sustainable Competitive Advantage	Business Model	“A good business model can create sustainable competitive advantages. By providing the vital link between a firm's vision and strategy with its organizational structures and processes, the business model determines the way a firm defines objectives, motivates effort, coordinates activities and allocates resources, as well as its sources of revenue, cost structure, and make-or-buy options.” [49]
		Competitive Advantage Concept	Regarding the “competitive advantage” concept, again, some subjective definition limitations arise due to its “latent” nature, where many literature definitions coexist with different meanings under different contexts, together with two other research streams, namely: the first defining competitive advantage in terms of performance (using proxies like ROI, ROE, market value) and the second defining it as its sources of determinants (depending on differentiation, location, technology, and a set of idiosyncratic resources and capabilities).” [48]
		Industry-Specific IT	“Industry-specific IT resources are valued because they reduce costs, supporting sustainable competitive advantages as a result.” [6]
		Sustainable Performance and Sustainable Competitive Advantage	“The objective of companies is to establish sustainable performance and sustainable competitive advantage by integrating technology into the decision-making process with corporate strategy. Businesses are supposed to be more flexible and responsive to strategic decision-making in the current dynamic environment. Companies that will maintain their competitive advantage are able to exceed the remainders in the long run.” [47]
		Open Innovation	“The open innovation paradigm suggests that a holistic, cognitive approach to corporate governance, based on a regime of cooperation between internal and external resources for the creation of value, opens the possibility of redefining business models in which knowledge develops horizontally. This is achieved through the involvement of all the actors involved in the corporate ecosystem to achieve a long-term, sustainable competitive advantage.” [53]
		Technologies and Working methods	“Select the right technologies and working methods so that the new technology can enable people to do their work according to the new or re-engineered process and create a sustainable competitive advantage.” [52]
	Sustainable Development	Sustainable Development Goals	“Digital transformation will also reduce corruption and facilitate meeting the objectives of the United Nations Sustainable Development Goals and Agenda 2030. Moreover, the interest is on understanding and presenting the impact of digitalization initiatives in economic growth and on the achievement of the Sustainable Development Goals of the United Nations; or to document their digital transformation around sustainability, efficiency, and quality of life of people in those spaces.” [56]
	Sustainable Innovation	Open Innovation Theory	According to open innovation theory, a holistic cognitive approach should allow the company to exploit efficiently internal knowledge, and absorb external knowledge concerning the dynamic environment. On the other hand, innovation has been defined as a tool that “recombines existing knowledge in new ways”, highlighting the limits and potential of the organization's cognitive substrate to encourage development and sustainable innovations [53].

**Table A5:** Human Resources dimension

Dimension	Categories	Codes	Phrases/Excerpts
Human Resources	Employee Experience	Digital Literate	“The shift towards culture, mind-set and competences demands a focus on employees. If they are not digitally literate, it could be highly difficult to survive digital transformations. For example, some articles stresses about the increased use of digital co-workers called as chatbots, software that participate in the day-to-day activities of the company as an active and engaged member of the team they belong. However, if employees are not ready for these kinds of digital technology applications, employee experience practices will not achieve the goal of creating a personalized, compelling and memorable environment for employees. It will rather generate information overload and anxiety apply employees.” [58]
	Career Dynamics	Diversity in the IT Workforce	“HRM concern is the lack of diversity in the IT workforce. While the benefits of a diverse workforce are well-known, the career dynamics of individuals with identity-based differences are not well understood. The IT sector is perceived to be a high-pressure environment where it is difficult to keep up with specialized technical skills for long periods of time. This commitment conflicts with the work-life balance of older workers who prefer to spend more time with their families. The desire for a healthier work-life balance may explain why some individuals transition from a specialized field to more generic management positions later in their career. Ultimately, there exists an age bias in a workforce that deals with new technologies and innovative firms, which is predominant in the IT sector.” [57]

**Table A6:** Smart Cities dimension

Dimension	Category	Codes	Phrases/Excerpts
Smart Cities	Smart Production/Manufacturing	DT Coverage	“Digital transformation (DT) has recently attracted increasing interest from scholars, practitioners, governments, and information and communication technology suppliers to develop automatized, optimized, and sustainable concepts such as smart cities, smart health, smart homes, smart government, and smart production, also known as Industry 4.0 or Industrial Internet of Things (IIoT).” [45]
		Sustainable Smart Manufacturing	“Sustainable smart manufacturing has been advanced by digital technologies such as IoT, cyber-physical systems, cloud computing, AI, big data analytics, and digital twin.” [55]
		Digital Manufacturing	“Digital manufacturing as an extensive ecosystem that includes different value activities and business functions, specific technologies (e.g., 3D printing, cyber-physical systems, IoT, smart products, digital platforms, advanced robotics, cloud computing and data analytics, etc.) and definitions (e.g., additive manufacturing, digital fabrication, home fabrication, presumption, Industry 4.0, industrial internet, smart manufacturing, etc.) are here considered as constitutive elements of this context.” [60]
